# Supplementary material for: Anxiety among cancer patients: A cross-sectional study at The University of Gondar comprehensive specialized hospital
Source: PLoS One. 2025 Jan 29;20(1):e0316748. doi: 10.1371/journal.pone.0316748 (PMC11778787; doi:10.1371/journal.pone.0316748)
Supplement: Attachment — Submitted filename: Response to Reviewers.docx [file pone.0316748.s002.docx]

| **Date:13/10/2024** |  |
| --- | --- |

**A point-by-point response to editors and reviewers**

**Title: Anxiety among Cancer Patients: A Cross-Sectional Study at the University of Gondar Comprehensive Specialized Hospital**

**Manuscript number:** PONE-D-24-31541

**Subject: Submitting a revised version of the manuscript**

We would like to thank the reviewers and editors for sharing their views and novel scholarly experiences. The comments are very imperative and I strongly believe in improving the manuscript. The authors try to address all the comments raised by the reviewers line by line in the main document. The point-by-point responses for each of the comments, questions, and the revised manuscript are provided in the attached documents.

Thank you for considering our manuscript again

**Response to the academic editor**

**Dear Editor, we appreciate your valuable feedback and requirements, and we have addressed them in our responses as outlined below.**

Journal Requirements:

When submitting your revision, we need you to address these additional requirements.

1. Please ensure that your manuscript meets PLOS ONE's style requirements, including those for file naming. The PLOS ONE style templates can be found at

https://journals.plos.org/plosone/s/file?id=wjVg/PLOSOne_formatting_sample_main_body.pdf and<https://journals.plos.org/plosone/s/file?id=ba62/PLOSOne_formatting_sample_title_authors_affiliations.pdf>

**Author response**: The authors used the PLOS ONE formatting style in the revised manuscript.

2. Your ethics statement should only appear in the Methods section of your manuscript. If your ethics statement is written in any section besides the Methods, please move it to the Methods section and delete it from any other section. Please ensure that your ethics statement is included in your manuscript, as the ethics statement entered into the online submission form will not be published alongside your manuscript.

**Author response:** The ethics statement only appears in the Methods section of our manuscript.

3. We note that you have indicated that there are restrictions to data sharing for this study. For studies involving human research participant data or other sensitive data, we encourage authors to share de-identified or anonymized data. However, when data cannot be publicly shared for ethical reasons, we allow authors to make their data sets available upon request. For information on unacceptable data access restrictions, please see http://journals.plos.org/plosone/s/data-availability#loc-unacceptable-data-access-restrictions.

Before we proceed with your manuscript, please address the following prompts:

a) If there are ethical or legal restrictions on sharing a de-identified data set, please explain them in detail (e.g., data contain potentially identifying or sensitive patient information, data are owned by a third-party organization, etc.) and who has imposed them (e.g., a Research Ethics Committee or Institutional Review Board, etc.). Please also provide contact information for a data access committee, ethics committee, or other institutional body to which data requests may be sent.

b) If there are no restrictions, please upload the minimal anonymized data set necessary to replicate your study findings to a stable, public repository and provide us with the relevant URLs, DOIs, or accession numbers. Please see http://www.bmj.com/content/340/bmj.c181.long for guidelines on how to de-identify and prepare clinical data for publication. For a list of recommended repositories, please see https://journals.plos.org/plosone/s/recommended-repositories. You also have the option of uploading the data as Supporting Information files, but we would recommend depositing data directly to a data repository if possible.

Please update your Data Availability statement in the submission form accordingly.

**Author response:** According to the ethical review committee, the data contains potentially identifying characteristics as well as sensitive patient information and cannot be shared publicly. All data requests can be sent to the Director of the School of Medicine (Dr. Mulugeta Ayalew at mulugetaayalew336@gmail.com), or the Chair, the Ethical Review Board (prof. Abebe Muche at abemuche14@gmail.com).

**Reviewer #1:**

**Dear Reviewer, we appreciate the valuable comments, suggestions, and requirements you provided, and we have addressed them in our responses as outlined below.**

**Lines 28-29: How did you selected your study participant?**

**Author response:** Participants were selected using a systematic random sampling method with a skip interval of one. We included this sentence in the revised manuscript (L 88-90).

**Lines 32-33: Why not you included OR and CIs?**

**Author response:** The authors modified the revised manuscript by including OR and CIs in the abstract.

**Line 34: What is your reference to say high?**

**Author response: Dear reviewer, thank you**. The study found a high prevalence of anxiety among cancer patients, compared to the previous studies, the reason we said "a high prevalence" is to emphasize that the current study observed a greater percentage or rate of anxiety among cancer patients compared to earlier research. It suggests that the anxiety levels among cancer patients in the present study were more significant or more frequent than what was reported in previous studies. This could be due to factors like differences in sample size, methodology, population characteristics, or even changes in the healthcare environment.

**Lines 45-62: Your background is too short to really bring or describe the problem under consideration. Please try to discuss your background in chronological order focusing ion Ethiopian context.**

**Author response: Dear reviewer, thank you for your comment**. As per your request, the authors added ideas and discussed the background in chronological order focusing on the Ethiopian context.

**Lines 75-77: Why not you considered predictor factors while you calculating your sample size or double population formula?**

**Author response:** Dear reviewer, while we assessed factors associated with anxiety among cancer patients, predictor factors were not considered in the sample size calculation due to the simplicity of the study design. Our primary objective was to focus on estimating the prevalence of anxiety.

**Line 81: Cannot be inclusion criteria.**

**Author response: we removed it from the revised manuscript.**
**Lines 126-130: For how many days you gave the training? How you did these checkups?**

**Author response:** The quality of data was ensured through the training of data collectors, close supervision, and prompt feedback. The training, which lasted one day, included instruction on interview techniques in accordance with the prepared tool. The checkups were done every other day.

**Lines 134-135: Is it bi-variate or bi-variabele and multivariate or multivariable logistic regration? Which selection method did you use?**
**Author response: Thank you for the question, we** used bivariable/multivariable in the analysis, and now we mentioned it the revised manuscript.

**Lines 138-139: Have you checked for collinearity? If so what was the result? And what was the result of goodness of fit?**

**Author response: Dear reviewer,** for this study, the Hosmer and Lemeshow goodness-of-fit test was applied, yielding a value of 0.414. Multicollinearity was also assessed using the Variance Inflation Factor (VIF) and tolerance through a pseudo-linear regression analysis. All independent variables had VIF values less than 5 and tolerance values greater than 0.1, indicating the absence of multicollinearity (L143-146).

**Lines 148-149: You can put this in the bracket including CI.**

**Author response:** Amendment is done. Please refer to (L169-170)
**Line 163: I think these findings are too inflated?**

**Author response: Thank you for your observation. We understand how these findings might appear elevated at first glance**. In our evaluation, the higher figures may be due to

1. The prevalence rates reported were obtained using validated tools like the GAD-7, which is a standard instrument for assessing anxiety in clinical settings. We ensured to follow proper diagnostic criteria and a robust sample size to enhance the accuracy of the findings
2. The higher rates in our study could also be attributed to factors such as the severity of illness in our sample or the stage of follow-up during which the patients were assessed. Cancer follow-up visits can be a particularly stressful time for patients, heightening anxiety levels.

While these numbers may seem elevated, they reflect the real and significant psychological challenges faced by cancer survivors. These insights are critical for improving patient care and guiding future mental health interventions.

**Lines 177: What is the difference between RR and OR? How to interpret these two? Please read. Do think your interpretation correct?**

**Author response: thank you very much for your comment**. We rewrite it again please refer it L180-190 in the revised manuscript.
**Line 192: What is cotta sampling procedures?**

**Author response:** In the discussion section, we originally used the term 'cotta sampling procedure' as employed by the authors of a referenced article to compare our findings. However, to avoid ambiguity and enhance clarity, we replaced it with 'non-systematic random sampling' in the revised manuscript.

**Lines 228-234: For whom you recommending?**

**Author response: Thank you**. We now, in the revised manuscript put specific recommendations to the specific organization (L236-240).
**Line 236: What was your reference to say high?**

**Author response**: Dear reviewer, as we tried to elaborate in the question raised at the abstract section, The study found a high prevalence of anxiety among cancer patients, compared to the previous studies, the reason we said "a high prevalence" is to emphasize that the current study observed a greater percentage or rate of anxiety among cancer patients compared to earlier research. It suggests that the anxiety levels among cancer patients in the present study were more significant or more frequent than what was reported in previous studies. This could be due to factors like differences in sample size, methodology, population characteristics, or even changes in the healthcare environment.

**Lines 240-243: What is the difference between abbreviations and acronyms? Are these acronyms or Abbreviations?**

**Author response: thank you very much for your comment.** An abbreviation is a shortened version of a word or phrase, not necessarily pronounced as a word whereas an acronym is a specific type of abbreviation that forms a word from the initial letters of a phrase. Based on this definition we used both abbreviations and acronyms (L248).

**Lines 245-247: I thought it was a group work. So why I?**

**Author response: thank you very much for your comment.** We appreciate your comment. The authors amended the revised manuscript based on your recommendation.

**Additional comments**

**There are a lots of grammatical and sentence clarity errors. Please improve grammar and spelling errors. Again you have to include statement of declaration, conflict of interest, author contributions and funding statement. Unless it is incomplete manuscript. I will recommend to carefully read authors guideline.**

**Author response:** Dear review, thank you for your comment. The author edited the paper and made clear all the grammar and sentence clarity issues in the revised manuscript. The statement of declaration, conflict of interest, author contributions, and funding statement are revised according to the journal authors' submission guidelines.

**Reviewer #2:**

**Dear Reviewer, we appreciate the valuable comments, suggestions, and requirements you provided, and we have addressed them in our responses as outlined below.**

 Reviewer #2: Avery good study the study format is very clear, not very wordy I have just comments:

**-Comment 1: The abbreviations I prefer to be put at the beginning of the study, not at the end.**

**Authors’ response:** Dear Reviewer, Thank you for your valuable feedback. In accordance with the journal's author guidelines, the abbreviations are placed after the conclusion section. We appreciate your understanding, and we will ensure that all formatting aligns with the journal's requirements.

**Comment 2: References: I wish to cite more recent references (2024/2023/2022).**

**Authors’ response:** Dear Reviewer, Thank you for your insightful comment. While we acknowledge the importance of using recent citations, we chose to include certain references because they provide fundamental insights and key findings that remain highly relevant to our study. These sources were carefully selected for their contribution to the field and the strength of their findings, which we believe add significant value to our work.
