# Supplementary material for: Anxiety among cancer patients: A cross-sectional study at The University of Gondar comprehensive specialized hospital
Source: PLoS One. 2025 Jan 29;20(1):e0316748. doi: 10.1371/journal.pone.0316748 (PMC11778787; doi:10.1371/journal.pone.0316748)
Supplement: Attachment — Submitted filename: Response_to_Reviewers_auresp_2.docx [file pone.0316748.s003.docx]

| **Date:27/11/2024** |  |
| --- | --- |

**A point-by-point response to editors and reviewers**

**Title: Anxiety among Cancer Patients: A Cross-Sectional Study at the University of Gondar Comprehensive Specialized Hospital**

**Manuscript number:** PONE-D-24-31541

**Subject: Submitting a revised version of the manuscript**

We would like to thank the reviewers and editors for sharing their views and novel scholarly experiences. The point-by-point responses for each of the comments, questions, and the revised manuscript are provided in the attached documents.

Thank you for considering our manuscript again

**Response to the academic editor**

Journal Requirements:

Please review your reference list to ensure that it is complete and correct. If you have cited papers that have been retracted, please include the rationale for doing so in the manuscript text or remove these references and replace them with relevant current references. Any changes to the reference list should be mentioned in the rebuttal letter that accompanies your revised manuscript. If you need to cite a retracted article, indicate the article’s retracted status in the References list and include a citation and full reference for the retraction notice.

**Author response:** Dear Editor, we reviewed the references, and they are complete and correct. We also declare that we did not cite papers that have been retracted.

**Response to Reviewer #1:**

Reviewer #1: I commented on this paper in a previous review. There are certain issues I recognized while I was checking for whether my first review comments were addressed or not. something like sampling technique, which cannot be corrected after data collection has been changed. For example, the author reported the "QOUTA" sampling technique in a previous submission and now reports it as a "systematic sampling technique. This is unacceptable!

**Author response:** Dear reviewer, Thank you for your feedback. We would like to clarify that we did not change our sampling technique. However, we added the following sentence to our manuscript: “Participants were selected using a systematic random sampling method with a skip interval of one” to directly address your previous question: “How did you select your study participants?”

Regarding the term “cotta sampling,” this is unrelated to our sampling technique. Originally, in the discussion section, we used the term ‘cotta sampling procedure’ as it was employed by the authors of a referenced article to compare their findings on the prevalence of anxiety among cancer patients with ours. In response to your question, “What is cotta sampling procedure?”, we replaced it with the term ‘non-systematic random sampling’ in the revised manuscript to ensure clarity.

We hope this explanation addresses your concerns. Please let us know if further clarification is needed.

**Reviewer #2:**

 Reviewer #2: Thank you for the prompt answer and adequate reply to all the comments given and wish to continue further research

**Authors’ response:** Dear Reviewer, Thank you for your thoughtful comments and feedback. We are pleased to hear that you are satisfied with our responses.
