# Supplementary material for: Anxiety among cancer patients: A cross-sectional study at The University of Gondar comprehensive specialized hospital
Source: PLoS One. 2025 Jan 29;20(1):e0316748. doi: 10.1371/journal.pone.0316748 (PMC11778787; doi:10.1371/journal.pone.0316748)
Supplement: Attachment — Submitted filename: Reviewer comments.docx [file pone.0316748.s001.docx]

**Reviewer’s comments**

Lines 28-29: How did you selected your study participant?

Lines 32-33: Why not you included OR and CIs?

Line 34: What is your reference to say high?

Lines 45-62: Your background is too short to really bring or describe the problem under consideration. Please try to discuss your background in chronological order focusing ion Ethiopian context.

Lines 75-77: Why not you considered predictor factors while you calculating your sample size or double population formula?

Line 81: Cannot be inclusion criteria.

Lines 126-130: For how many days you gave the training? How you did these checkups?

Lines 134-135: Is it bi-variate or bi-variabele and multivariate or multivariable logistic regration? Which selection method did you use?

Lines 138-139: Have you checked for collinearity? If so what was the result? And what was the result of goodness of fit?

Lines 141-144: You put this statement in the wrong place according to author guidelines. Please refer to author guidelines of the journal.

Lines 148-149: You can put this in the bracket including CI.

Line 163: I think these findings are too inflated?

Lines 177: What is the difference between RR and OR? How to interpret these two? Please read. Do think your interpretation correct?

Line 192: What is cotta sampling procedures?

Lines 228-234: For whom you recommending?

Line 236: What was your reference to say high?

Lines 240-243: What is the difference between abbreviations and acronyms? Are these acronyms or Abbreviations?

Lines 245-247: I thought it was a group work. So why I?

**Additional comments**

There are a lots of grammatical and sentence clarity errors. Please improve grammar and spelling errors. Again you have to include statement of declaration, conflict of interest, author contributions and funding statement. Unless it is incomplete manuscript. I will recommend to carefully read authors guideline.
